# Human Soluble Recombinant Thrombomodulin, ART-123, Resolved Early Phase Coagulopathies, but Did Not Significantly Alter the 28 Day Outcome in the Treatment of DIC Associated with Infectious Systemic Inflammatory Response Syndromes

**DOI:** 10.3390/jcm8101553

**Published:** 2019-09-27

**Authors:** Shusuke Mori, Tomohiko Ai, Toshiki Sera, Kanae Ochiai, Yasuhiro Otomo

**Affiliations:** Trauma and Acute Critical Care Center, Tokyo Medical and Dental University, 1-5-45 Yushima, Bunkyo-ku, Tokyo 113-8510, Japan

**Keywords:** sepsis, systemic inflammatory response syndrome, disseminated intravascular coagulation, recombinant thrombomodulin, ART-123

## Abstract

Disseminated intravascular coagulation (DIC) is a catastrophic systemic disorder of coagulation, resulting in uncontrollable bleeding, multiple organ failure, and death. Sepsis is one of the common causes of DIC. Despite many attempts to correct these coagulation pathologies, no adjunctive treatments have been shown to improve the mortality of DIC associated with sepsis. Although some clinical studies showed a recently developed human recombinant thrombomodulin, ART-123, might be effective in the treatment of DIC, few randomized, placebo-controlled studies have been conducted. In this study, we treated 60 DIC patients associated with systemic inflammatory response syndrome (SIRS) using ART-123 (*n* = 29) or saline as a placebo (*n* = 31). The basal clinical characteristics were similar in both groups. We compared clinical severity scores and DIC score in acute phase, and 28 day mortality between the two groups. Our study demonstrated the DIC score improved a few days earlier in the ART-123 group than the placebo group, and there were no major life-threatening adverse events in both groups. The overall survival rate at day 28 was not significantly altered. In conclusion, ART-123 can be used safely in DIC associated with infectious SIRS patients; however, its true efficacy in the treatment of DIC needs to be further investigated.

## 1. Introduction

Disseminated intravascular coagulation (DIC) is a catastrophic systemic disorder of coagulation, resulting in uncontrollable bleeding, multiple organ failure (MOF) and death. It can be caused by various medical conditions including sepsis, trauma, cancers, obstetrical complications, vascular disorders, toxins, immunologic disorders, and inflammations [1]. Excessive thrombin production triggered by overexpressed tissue factor associated with inflammation, activated monocytes, and vascular lesion ignites the explosive chain reaction in platelet activation, consumption of natural coagulation inhibitors, and inhibition of fibrinolysis, leading to a hypercoagulable state, deposition of fibrin in micro vessels, and hemorrhage [2].

Bacterial infections are most frequently associated with the development of DIC. Clinically overt DIC may occur in up to 50% of patients with sepsis associated with gram-negative bacteria [1]. The mortality of sepsis complicated with DIC might be significantly higher than sepsis without DIC [3]. Therefore, it is medically relevant to manage DIC in patients with sepsis. International and Japanese guidelines for the treatment of DIC state that treatment of underlying diseases is the most effective way to reverse this catastrophic coagulation disorder [4,5,6]. However, there are some discrepancies between the Japanese DIC management guidelines and other guidelines mainly regarding supportive adjunctive treatments aiming at coagulation disorders. The use of soluble human recombinant thrombomodulin is one of those discrepancies [7]. Human thrombomodulin binds to thrombin to form a complex that inhibits thrombin activity, which converts protein C into its activated form allowing it to blind to surface receptors of vascular endothelial cells. Activated protein C plays an important role in suppressing coagulation and inflammatory systems [8].

The effects of human soluble recombinant thrombomodulin, ART-123, in the treatment of DIC have been examined in several clinical trials. In a phase III, randomized, double-blind study, ART-123 reduced the bleeding events in patients with DIC-associated hematological malignancy or infectious diseases compared to treatment with heparin [9]. However, the sub-analysis of this clinical trial did not show the efficacy of ART-123 in the DIC associated with infectious diseases [10]. In a phase IIb, international, double-blind, randomized, placebo-controlled trial, the efficacy of ART-123 was tested on 371 septic patients with suspected DIC defined by impaired platelet count and prolonged prothrombin time based upon the DIC score by the International Society on Thrombosis and Hemostasis (ISTH). They concluded that ART-123 did not cause any adverse events, but there were no significant differences in 28-day mortality between ART-123 and placebo groups [11]. The Sepsis Coagulopathy Asahi Recombinant LE Thrombomodulin (SCARLET) randomized clinical trial, an expanded phase III clinical trial of the phase IIb study, also failed to show the improvement of thrombomodulin in the 28-day all-cause mortality in the treatment of sepsis-associated DIC [12]. On the contrary, recent retrospective observational studies in Japan claimed that ART-123 might be beneficial to treat sepsis-induced DIC in patients with severe coagulopathy [13] or severe respiratory failure [14].

Although ART-123 has been widely used as an adjunctive therapy in Japan, it is still unclear whether it is truly effective in improving the outcome of DIC. It is difficult to interpret the results in previous clinical trials due to different study designs: prospective or retrospective; different control groups (no optional treatments or heparin); different diagnostic criteria (ISTH or Japanese Association for Acute Medicine (JAAM)); and different parameters to evaluate outcomes (bleeding, DIC score, or mortality) [3,9,11,14,15]. Thus, we propose that more data needs to be collected and evaluated in Japanese populations who might have originated from East Asia. In this prospective, randomized, placebo-controlled study, we investigated the effects of ART-123 in the treatment of Japanese patients with DIC associated with infectious systemic inflammatory response syndrome (SIRS) including surgical treatments at our emergency center in the past eight years.

## 2. Materials and Methods

### 2.1. Ethical Guidelines

This study was conducted in compliance with good clinical practice and the ethical principles of the Declaration of Helsinki. Prior approval was obtained from the ethics review boards of Tokyo Medical and Dental University (TMDU) (M2000-1314). Written informed consent was obtained from all patients or acceptable representatives.

### 2.2. Subjects

Patients with infectious disease-associated SIRS with acute DIC were recruited. SIRS was judged by SIRS score (Table 1), and infectious diseases were judged by the attending physicians based upon physical examinations, imaging studies, and laboratory examinations. DIC was diagnosed according to the diagnostic criteria established by the Japanese Association for Acute Medicine (JAAM DIC criteria; Table 2) [16]. Inclusion criteria were as follows: (i) a DIC score of more than 4 points; (ii) 18 years old or older; and (iii) patients requiring hospital admission or inpatient status. Table S1 shows the International Society on Thrombosis and Hemostasis for a comparison to JAAM score [5].

Exclusion criteria were as follows: (i) fatal or life-threatening bleeding (intracranial, gastrointestinal, or pulmonary bleeding); (ii) dialysis therapy for renal failure or extracorporeal circulation for blood purification; (iii) pregnancy, nursing, or potential pregnancy; (iv) minors; and (v) patients judged as inappropriate at the discretion of attending physicians.

### 2.3. Treatment Assignments

Between October 2008 and July 2015, a total of 62 patients with DIC associated with sepsis were enrolled. Sepsis was defined by SIRS criteria throughout the study [17]. After random allocation by sealing numbers, 29 patients were assigned to recombinant thrombomodulin, ART-123 group, and 31 patients were assigned to the control group. Patients in the ART-123 group received a drip infusion of ART-123 (0.06 mg/kg/30 min) for 6 consecutive days. The control group received a drip infusion of saline (100 mL) for 30 min once daily. During the infusion of the study drug, the use of drugs that might affect drug efficacy such as anticoagulants (including synthetic protease inhibitors), antiplatelet agents, and fibrinolytic agents, was not administered. Patients were followed for 10 days post-infusion.

### 2.4. Evaluation of Patients

The patients’ clinical severity scores were evaluated everyday between day 1 and 10 using the sequential organ failure assessment score (SOFA) [18,19] and acute physiology, age, chronic health evaluation II (APACHE II) [20,21].

The prospectively defined primary efficacy endpoint was DIC resolution rate as assessed at 10 days after the start of infusion. In accordance with JAAM DIC criteria (Table 2), DIC scores were determined daily before drug infusion, and DIC resolution rate was assessed. Resolved DIC status was defined on the basis of DIC scores ≤4 points. Secondary endpoints included clinical course of bleeding symptoms and mortality at 28 days after the start of infusion.

Coagulation tests were conducted on the day of registration, at baseline, and drug infusion to measure the following: fibrin and fibrinogen degradation products (FDP); fibrinogen; prothrombin time international-normalized ratio (PT-INR); platelet count; antithrombin; and D-dimer. All adverse events observed until 28 days post-infusion, including new or exacerbated bleeding and organ symptoms, and abnormal changes in clinical laboratory test findings, were recorded by the investigators. Serious adverse events, including death, life-threatening events, prolonged hospitalization, permanent or significant disorder or dysfunction, or other severe medical events, were recorded throughout the study period.

### 2.5. Statistics

The data for clinical parameters including APACHE II, SOFA, and DIC scores and laboratory data were compared between ART-123 and control groups using repeated measure ANOVA. Data comparison between the two groups was performed by the Mann–Whitney U test, Chi-squared test, or Student *t*-test where appropriate. The survival curves were plotted with Kaplan–Meier estimator (SPSS, IBM, New York, NY, USA).

## 3. Results

### 3.1. Baseline Characteristics

Table 3 summarizes the baseline clinical characteristics of the patients (day 1). Patients’ demographics and medical conditions on admission were comparable between ART-123 and the control groups: APACHE II [21], SOFA [22], and JAAM DIC scores [16]. The platelet counts, C-reactive protein (CRP), and coagulation parameters on admission were also comparable between the two groups. Nine cases of ART-123 group and 13 cases of the control group were surgically treated to control the infectious causes. Most of the cases were associated with infectious peritonitis due to perforated gastrointestinal tracts, and repair surgeries were performed (Table 4). There was no significant difference in these clinical characteristics between the two groups.

All patients were treated to stabilize hemodynamic and respiratory state, and broad spectrum antibiotics were administered until the accountable microorganisms were detected by blood cultures. 

### 3.2. Overall General Clinical Conditions

**Figure 1:**
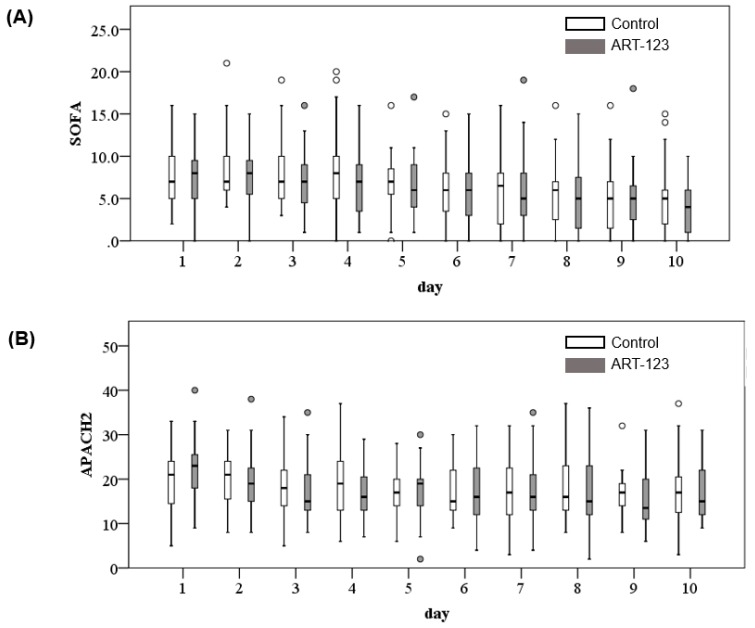
Changes of time course in SOFA (**A**) and APACHE II (**B**) scores from day 1 to day 10.

Figure 1 shows the changes of time course in SOFA and APACHE II scores from day 1 to day 10. While APACHE II scores were not drastically improved, SOFA scores were slightly improved at day 10 compared to the day 1. However, there was no difference between the control and ART-123 groups (SOFA: *p* = 0.600, Figure 1A; APACHE II: *p* = 0.868, Figure 1B).

**Figure 2:**
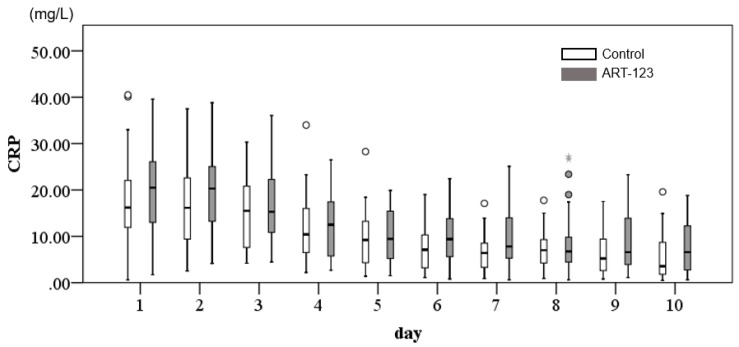
Changes of time course in C-reactive protein (CRP) from day 1 to day 10.

As a marker of inflammation, CRP values were measured from day 1 to day 10. Although CRP values were significantly improved in both groups at day 10, there was no significant difference between the ART-123 and control groups (*p* = 0.071, Figure 2).

### 3.3. Change of DIC Parameters

**Figure 3:**
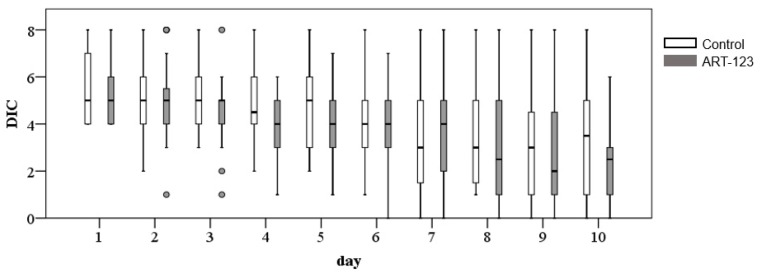
Changes of time course in DIC scores from day 1 to day 10.

**Figure 4:**
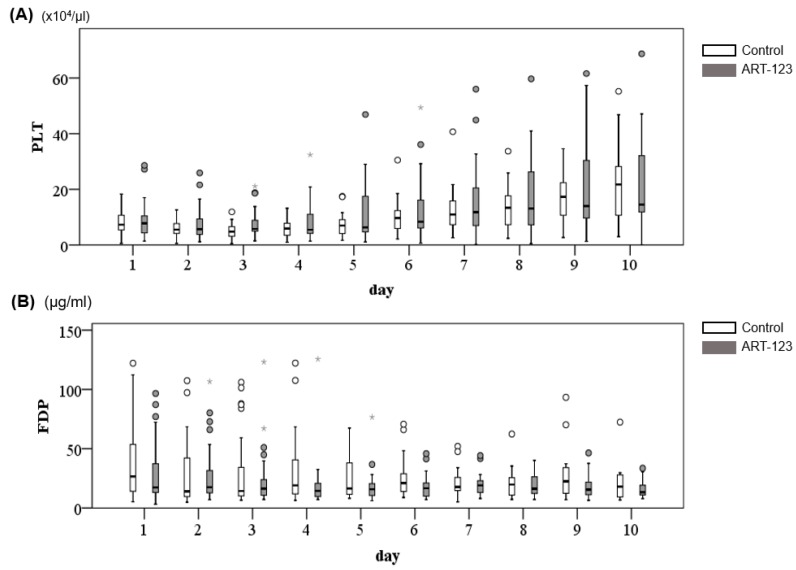
Changes of time course in platelets (PLT) (**A**) and fibrin/fibrinogen degradation products (FDP) (**B**).

Figure 3 shows the time course of DIC scores. While the DIC scores were improved in 10 days in both groups, the DIC scores at day 3 and 4 were significantly lower in ART-123 than the control group (*p* < 0.05). This indicates ART-123 might promote DIC resolution. Platelet and FDP counts were also improved in 10 days in both groups; however, there was no significant difference between the two groups (platelet: *p* = 0.851, Figure 4A; FDP: *p* = 0.425, Figure 4B).

**Figure 5:**
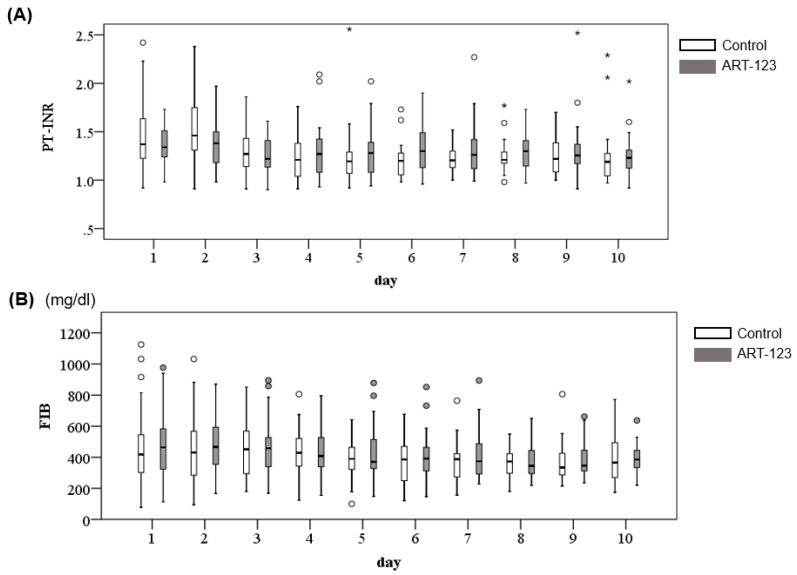
Changes of time course in prothrombin time-international normalized ratio (PT-INR) (**A**) and fibrinogen (FIB) (**B**).

The PT-INR values were slightly prolonged for all patients at day 1 and were not significantly altered over the 10 days from admission (*p* = 0.302, Figure 5A). Although fibrinogen was not included to determine the JAAM DIC score, it was also monitored and there was no significant change over the 10 days (Figure 5B). Since all patients showed fibrinogen <1.0 g/L, it did not affect the ISTH score.

### 3.4. Overall Mortality

**Figure 6:**
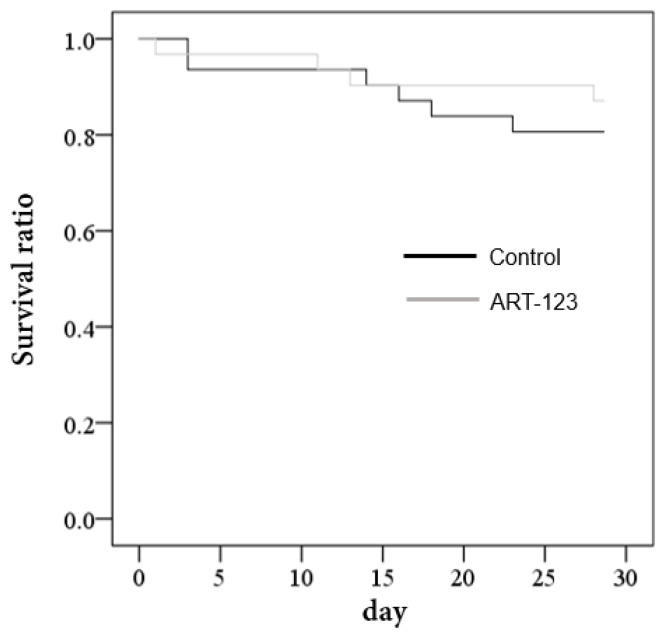
Kaplan–Meier plot of survival rate from admission to day 28.

Figure 6 shows the Kaplan–Meier plot of survival rate from admission to day 28. The overall mortality rate at day 28 was not significantly different between the two groups (*p* = 0.510). There was no significant difference in terms of the severity of clinical conditions (APACHE II and SOFA scores), and DIC scores and DIC resolution rate on day 28 (Table 5).

Table 6 summarizes the clinical characteristics of the deceased cases. All deceased cases were the cases that were not treated by surgical procedures. The APACHE II, DIC scores, and CRP values were comparable between the two groups (*p* > 0.05). The causes of death were worsening of sepsis leading to multiple organ failure in both groups.

## 4. Discussion

This study is one of the few prospective, randomized, placebo-controlled studies to investigate the effects of human recombinant thrombomodulin, ART-123, in the treatment of DIC associated with infectious systemic inflammatory response syndrome (SIRS). A total of 60 patients who presented SIRS-associated DIC were randomly assigned to ART-123 (*n* = 29) or the placebo group (*n* = 31), and clinical outcomes were compared between the two groups. Our data demonstrated that ART-123 did not significantly improve the mortality at 28 hospital-days although the DIC score was resolved a few days earlier in the treated group than the placebo group.

The pathology of DIC has been known since the 1990s [1], and, to date, there is a consensus that treatment of underlying diseases is the most effective way to resolve DIC without any additional treatments [2,7]. However, in reality, additional available therapies such as blood transfusion, fresh frozen plasma, anticoagulants, and/or antithrombotics have been used in attempts to control life-threatening bleeding and coagulation disorders even though those treatments might not be meaningful. This dilemma is similar to that of the cardiac arrhythmia suppression trial (CAST). Arrhythmia can be life-threatening after myocardial infarction, and cardiologists thought suppressing frequent premature ventricular contractions after a heart attack using antiarrhythmics could improve mortality. Ironically, the mortality rate significantly increased with antiarrhythmics despite the reduction of premature ventricular contractions, and the trial was discontinued [23]. Based upon recent clinical trials, including ours, ART-123 may not be harmful, but it does have a substantial impact on medical expenses.

Efficacy and safety of optional therapies for DIC aiming at correcting coagulation disorders have been examined in many clinical studies. Disappointingly, no treatments have been proven, so far, to benefit the patients. In some studies, those therapies have even worsened the mortality or caused adverse events. These include: antithrombin concentrates (KyberSept trial) [24]; activated protein C (PROWLESS trial) [25]; Drotecognin Alfa (ADDRESS and PROWLESS-SHOCK trials) [26,27]; tissue factor pathway inhibitor (OPTIMIST trial) [28]; and anticoagulants [29,30]. ART-123 is a relatively new therapy aimed at restoring deleterious coagulation. Despite several clinical studies including randomized, double-blind trials, definitive conclusions have not yet been made [3,9,11].

In a multicenter, randomized, double-blind clinical study, Saito et al. reported that human soluble thrombomodulin could resolve the DIC by 16% compared to the group treated with heparin, but failed to significantly improve mortality of DIC [9]. However, more than half of the 241 enrolled patients were suffering from hematology malignancy, and the DIC resolution rate was higher in the patients with malignancy compared to patients with infectious diseases [9]. In an international multicenter, randomized, double-blind, clinical trial, Vincent et al. studied the effects of ART-123 on the 28-day mortality rate and DIC resolution in 370 patients with sepsis and suspected DIC compared to 370 patients treated with a placebo. This study, also, failed to observe a significant difference in the 28-day mortality, the resolution of DIC, and changes in inflammatory markers between the two groups [11]. A recent retrospective study using a large medical database in Japan (Medical Data Vision Co., Ltd.) demonstrated that ART-123 treatment for Japanese postsurgical patients with DIC was associated with significantly fewer bleeding-related adverse events compared with those receiving other DIC treatments; however, it failed to show the difference in the overall mortality [31]. The SCARLET randomized clinical trial [12], a phase III clinical trial following the previous phase IIb clinical trial [11] was just published in 2019. In the trial, they reported that the incidence of life-threatening major bleeding was less in the thrombomodulin group than the placebo group (5.8% vs. 4.0%). However, the 28-day all-cause mortality was not significantly different.

Our data, one of the few prospective randomized trials in Asians, corroborated the results of these clinical trials. However, our data demonstrated that resolution of DIC took place 2–3 days earlier in the ART-123 group than the control group although the PT-INR in our patients was relatively lower than the SCARLET trial. In addition, severe life-threatening bleeding events were not observed in either groups. Importantly, most of the death were associated with uncontrollable infection and multiple organ failures (Table 6). Thus, our study indicates that ART-123 might play a role to resolve the DIC associated with SIRS.

Moving forward the question is: “Should we still continue to examine the effects of thrombomodulin in this entity of patients?” Despite millions of patients who have been suffering from DIC associated with sepsis, the details of pathophysiological mechanism remain unknown [32]. Considering the number of patients, very few studies have been performed to examine the effects of reagents aiming at specific targets rather than supporting therapies. This might be due to the complex pathophysiology of the diseases. There are too many potential targets in the cascades and pathways of coagulation systems [33] and sepsis [34] to elucidate what is the true target(s) to prevent multi-organ failure. Furthermore, in order to compare the pharmacological effects in a stringent manner, the conditions of two groups (i.e., treated and control patients) have to be identical, including genetic backgrounds. However, achieving those conditions are highly improbable since even monozygotic twins have almost no chance of suffering from the exact same type of sepsis-induced DIC. Therefore, at a minimum, it is necessary to accumulate further data on patients with very similar conditions using consistent evaluation methods and clinical outcomes.

Our study has several significant limitations. This is a single center study with a relatively small number of patients although it is a randomized, placebo controlled, study. In the SCARLET trial, the sample size of 800 patients was supposed to provide 80% power at a 5% two-sided α level based upon a reduction level of 8% in mortality derived from the phase IIb clinical study [11,12]. However, the SCARLET trial did not show significant difference in the 28-day mortality between ART-123 and the placebo group [12]. In a phase III, randomized, double-blind clinical trial to compare the effects of ART-123 and heparin in Japanese DIC patients, 110 patients were thought to be required to provide 80% power at a 5% two-sided α level. However, the underlying diseases contained hematological malignancy and infectious diseases, and non-significant trends in favor of ART-123 were observed for mortality in infectious patients compared to heparin [9]. In a Japanese retrospective cohort study to examine the effects of recombinant human thrombomodulin in sepsis-induced DIC patients, 12 patients were assigned to the treatment group and 23 patients were assigned to the control. The study showed that the seven-day DIC score was improved in the treatment group, but the 28-day mortality was not significantly different. In this study, a sample size of 114 patients was supposed to provide 90% power at a 5% two-sided α level for the mortality, and 105 patients for the seven-day DIC resolution [13]. In our study, according to Cohen [35], a sample size of 79 patients or 84 patients provided 80% power at a 5% two-sided α level for the mortality or the DIC resolution. Thus, our sample size was close to but smaller than the calculated values of the required sample size. When performing a randomized controlled trial, sample size should be carefully considered. To satisfy the required sample size, which sometimes can be substantial, it is often necessary to depend on multicenter trials. The merit of single center trials is that the condition of the treatment can be consistent and access to detailed patient records is very useful to analyze the reasons why a phenomenon is observed. However, there was a significant difference in the timing for resolution of DIC scores, which, in our opinion, makes our study meaningful.

The medical conditions among the patients vary in terms of the primary infectious sites, causes, severity of sepsis, hemodynamics, immunity, type of antibiotics, and drug-metabolisms. However, these limitations are similar to the issues observed in previous clinical trials. Also, most of the patients in this study were enrolled based upon their SIRS score, although we evaluated SOFA and APACHE II scores as well. This was due to the fact that our study was performed before the recent change in the definition of sepsis [36]. Therefore, we cannot deny that changing the inclusion criteria based upon the new definition of sepsis might have affected the results. Another limitation is that it is impossible to evaluate effects of any reagents on DIC separately from underlying conditions (sepsis in this study). Since treatments of sepsis are supposed to resolve DIC, it is impossible to dissect what actually improves DIC. Thus, we cannot investigate the pathophysiology of DIC in the absence of the underlying diseases in this study.

## 5. Conclusions

Our study, one of the few prospective, randomized, placebo-control studies, investigated the efficacy and safety of human recombinant thrombomodulin, ART-123, in 60 patients with DIC associated with infectious SIRS (29 ART-123 vs. 31 placebo). Our study demonstrated that the JAAM DIC score was significantly improved a few days earlier in the ART-123 group compared to the placebo group. There were no major life-threatening bleeding events in both groups. The overall mortality at day 28 after admission was not significantly different. Thus, we conclude that ART-123 can be used safely in the DIC associated with infectious SIRS patients although further studies are warranted to investigate the true efficacy of this adjunctive therapy in DIC, and to dissect its pharmacological effects in various disease statuses.

## Figures and Tables

**Table 1:** Systemic inflammatory response syndrome (SIRS) score.

	Points
Temp >38 °C or <36 °C	1
Heart rate >90 beats per min (bpm)	1
Respiratory rate >20 bpm or PaCO2 <32 mmHg	1
White blood cells (WBCs) >12,000/mm^3^, <4000/mm^3^, or >10% bands	1
Meets SIRS definition	≥2

**Table 2:** Japanese Association for Acute Medicine (JAAM) score.

	Point
Underlying disease	0
Clinical symptoms (SIRS score ≥3)	1
Platelet count (×10^4^/µL)	
8–12 or 30% reduction in 24 h	1
<8 or >50% reduction in 24 h	3
Fibrin-related marker	
Fibrinogen degradation products (FDPs) (µg/mL)	
10≤ but <25	1
≥25	3
Fibrinogen (mg/dL)	N/A
Prothrombin time international-normalized ratio (PT-INR)	
≥1.2	1
Diagnosis of disseminated intravascular coagulation (DIC)	≥4

**Table 3:** Baseline clinical characteristics.

	ART-123 (*n* = 29)	Placebo (*n* = 31)	*p*-Value	
Age, years	72.9 ± 14.1	75.5 ± 11.0	0.416	†
Gender (M:F)	15:14	15:16	0.667	††
Severity score				
APACHE II score	23.0 (18.0; 26.0)	21.0 (14.0; 24.0]	0.217	†††
SOFA score	8.0 ± 3.8	7.8 ± 3.4	0.779	†
DIC score	5.1 ± 1.2	5.7 ± 1.5	0.092	†
Laboratory data				
Platelet (×10)	8.8 ± 6.3	9.3 ± 7.9	0.810	†
FDP	18.3 (13.1; 56.7)	26.8 (14.3; 79.7)	0.260	†††
Fibrinogen	464.0 (348.0; 588.0)	418.0 (294.0; 562.0)	0.673	†††
PT-INR	1.3 (1.2; 1.5)	1.4 (1.2; 1.6)	0.709	†††
Antithrombin	39.1 (30.9; 52.5)	34.4 (27.5; 44.7)	0.478	†††
CRP	20.5 (12.8; 26.1)	16.2 (10.7; 22.9)	0.278	†††
Primary infection site				
Lung	8	8	1.000	
Digestive system	7	7	1.000	
Bile duct	3	3	1.000	
Soft tissue	1	3	0.614	
Kidney/urinary tract	3	5	0.710	
Liver	3	1	0.338	
Others	2	2	1.000	
Unknown	2	3	1.000	
Surgery	9 (31%)	13 (42%)	0.797	††

†: *t*-test, ††: Chi-squared test, †††: Mann–Whitney U test. APACHE II: acute physiology, age, chronic health evaluation II; SOFA: sequential organ failure assessment score; FDP: fibrin/fibrinogen degradation products; PT-INR: prothrombin time-international normalized ratio; CRP: C-reactive protein.

**Table 4:** Surgical treatments.

	ART-123 (*n* = 9)	Control (*n* = 13)
Gastrointestinal tract	5	7
Bile duct		2
Renal	1	3
Abdominal cyst	1	
Vascular graft	1	
Soft tissue	1	1

**Table 5:** Outcome.

	ART-123 (*n* = 29)	Control (*n* = 31)	*p*-Value
28 day mortality	7	7	0.56
Adverse event	1	0	
APACHE II score	14	17	0.26
SOFA score	4.8	5	0.44
DIC score	2	3.5	0.36
DIC resolution	21	19	0.26

**Table 6:** Deceased cases.

ID	Treatment	Age	Gender	Underlying disease	Surgery	APACHE II	CRP	DIC	Day	Cause
1	ART123	82	M	Pneumonia	None	24	26.1	4	9	Pneumonia/sepsis
5	ART123	67	F	AAA graft infection	None	26	11.9	4	50	AAA graft infection/sepsis
7	ART123	71	F	Pneumonia/cholangitis	None	24	26.9	4	44	Pneumonia/MOF
17	ART123	75	F	Unknown	None	16	12.8	5	47	Pneumonia/ARDS
22	ART123	81	F	Intestine/strangulation	None	27	24.5	4	26	ARDS/sepsis
41	ART123	87	F	Pneumonia	None	23	20.8	4	12	Pneumonia/sepsis
51	ART123	77	M	Brain abscess/miliary TB	None	33	20.6	4	4	Tuberculosis/sepsis
8	Control	84	F	Pneumonia	None	26	29.4	8	13	Pneumonia/sepsis (autopsy)
16	Control	74	M	Cholecystitis/peritonitis	None	30	14.9	6	17	Sepsis
20	Control	83	F	Heat stroke	None	24	15.9	7	14	Sepsis
23	Control	81	F	Pneumonia	None	24	5.9	4	22	Pneumonia/ARDS
35	Control	45	F	Neck abscess	None	29	33	8	32	Sepsis
47	Control	72	F	Infectious endocarditis	None	25	40.5	8	41	IE/sepsis
51	Control	72	F	Unknown *	None	33	14.7	6	2	Sepsis

AAA: abdominal aortic aneurysm; ARDS: acute respiratory distress syndrome; TB: tuberculosis; IE: infectious endocarditis; MOF: multiple organ failure; * autopsy.

## Supplementary Materials

The following are available online at https://www.mdpi.com/2077-0383/8/10/1553/s1, Table S1: DIC score by the International Society on Thrombosis and Hemostasis.

## References

[1] Levi M., Ten Cate H. (1999). Disseminated intravascular coagulation. N. Engl. J Med..

[2] Papageorgiou C., Jourdi G., Adjambri E., Walborn A., Patel P., Fareed J. (2018). Disseminated Intravascular Coagulation: An Update on Pathogenesis, Diagnosis, and Therapeutic Strategies. Clin. Appl. Thromb. Hemost..

[3] Hayakawa M., Yamakawa K., Saito S., Uchino S., Kudo D., Iizuka Y. (2016). Recombinant human soluble thrombomodulin and mortality in sepsis-induced disseminated intravascular coagulation. A multicentre retrospective study. Thromb. Haemost..

[4] Levi M., Toh C.H., Thachil J., Watson H.G. (2009). Guidelines for the diagnosis and management of disseminated intravascular coagulation. British Committee for Standards in Haematology. Br. J. Haematol..

[5] Taylor F.B., Toh C.H., Hoots W.K., Wada H., Levi M. (2001). Towards definition, clinical and laboratory criteria, and a scoring system for disseminated intravascular coagulation. Thromb. Haemost..

[6] Wada H., Asakura H., Okamoto K., Iba T., Uchiyama T., Kawasugi K. (2010). Expert consensus for the treatment of disseminated intravascular coagulation in Japan. Thromb. Res..

[7] Levi M. (2010). Japanese consensus for disseminated intravascular coagulation (DIC): Is it a small world after all?. Thromb. Res..

[8] Kisiel W. (1979). Human plasma protein C: Isolation, characterization, and mechanism of activation by alpha-thrombin. J. Clin Investig..

[9] Saito H., Maruyama I., Shimazaki S., Yamamoto Y., Aikawa N., Ohno R. (2007). Efficacy and safety of recombinant human soluble thrombomodulin (ART-123) in disseminated intravascular coagulation: Results of a phase III, randomized, double-blind clinical trial. J. Thromb. Haemost..

[10] Aikawa N., Shimazaki S., Yamamoto Y., Saito H., Maruyama I., Ohno R. (2011). Thrombomodulin alfa in the treatment of infectious patients complicated by disseminated intravascular coagulation: Subanalysis from the phase 3 trial. Shock.

[11] Vincent J.L., Ramesh M.K., Ernest D., LaRosa S.P., Pachl J., Aikawa N. (2013). A randomized, double-blind, placebo-controlled, Phase 2b study to evaluate the safety and efficacy of recombinant human soluble thrombomodulin, ART-123, in patients with sepsis and suspected disseminated intravascular coagulation. Crit. Care Med..

[12] Vincent J.L., Francois B., Zabolotskikh I., Daga M.K., Lascarrou J.B., Kirov M.Y. (2019). Effect of a Recombinant Human Soluble Thrombomodulin on Mortality in Patients with Sepsis-Associated Coagulopathy The SCARLET Randomized Clinical Trial. JAMA-J. Am. Med. Assoc..

[13] Kato T., Matsuura K. (2018). Recombinant human soluble thrombomodulin improves mortality in patients with sepsis especially for severe coagulopathy: A retrospective study. Thromb. J..

[14] Yoshihiro S., Sakuraya M., Hayakawa M., Ono K., Hirata A., Takaba A. (2018). Recombinant Human Soluble Thrombomodulin Contributes to Reduced Mortality in Sepsis Patients with Severe Respiratory Failure: A Retrospective Observational Study Using a Multicenter Dataset. Shock.

[15] Yamakawa K., Ogura H., Fujimi S., Morikawa M., Ogawa Y., Mohri T. (2013). Recombinant human soluble thrombomodulin in sepsis-induced disseminated intravascular coagulation: A multicenter propensity score analysis. Intensive Care Med..

[16] Wada H., Gabazza E.C., Asakura H., Koike K., Okamoto K., Maruyama I. (2003). Comparison of diagnostic criteria for disseminated intravascular coagulation (DIC): Diagnostic criteria of the International Society of Thrombosis and Hemostasis and of the Japanese Ministry of Health and Welfare for overt DIC. Am. J. Hematol..

[17] Bone R.C., Balk R.A., Cerra F.B., Dellinger R.P., Fein A.M., Knaus W.A. (1992). Definitions for sepsis and organ failure and guidelines for the use of innovative therapies in sepsis. The ACCP/SCCM Consensus Conference Committee. American College of Chest Physicians/Society of Critical Care Medicine. Chest.

[18] Vincent J.L., de Mendonca A., Cantraine F., Moreno R., Takala J., Suter P.M. (1998). Use of the SOFA score to assess the incidence of organ dysfunction/failure in intensive care units: results of a multicenter, prospective study. Working group on “sepsis-related problems” of the European Society of Intensive Care Medicine. Crit. Care Med..

[19] Raith E.P., Udy A.A., Bailey M., McGloughlin S., MacIsaac C., Bellomo R. (2017). Prognostic Accuracy of the SOFA Score, SIRS Criteria, and qSOFA Score for In-Hospital Mortality Among Adults with Suspected Infection Admitted to the Intensive Care Unit. JAMA.

[20] Knaus W.A., Zimmerman J.E., Wagner D.P., Draper E.A., Lawrence D.E. (1981). APACHE-acute physiology and chronic health evaluation: A physiologically based classification system. Crit. Care Med..

[21] Knaus W.A., Draper E.A., Wagner D.P., Zimmerman J.E. (1985). APACHE II: A severity of disease classification system. Crit. Care Med..

[22] Vincent J.L., Moreno R., Takala J., Willatts S., De Mendonca A., Bruining H. (1996). The SOFA (Sepsis-related Organ Failure Assessment) score to describe organ dysfunction/failure. On behalf of the Working Group on Sepsis-Related Problems of the European Society of Intensive Care Medicine. Intensive Care Med..

[23] Cardiac Arrhythmia Suppression Trial I (1989). Preliminary report: effect of encainide and flecainide on mortality in a randomized trial of arrhythmia suppression after myocardial infarction. N. Engl. J. Med..

[24] Warren B.L., Eid A., Singer P., Pillay S.S., Carl P., Novak I. (2001). Caring for the critically ill patient. High-dose antithrombin III in severe sepsis: A randomized controlled trial. JAMA.

[25] Bernard G.R., Vincent J.L., Laterre P.F., LaRosa S.P., Dhainaut J.F., Lopez-Rodriguez A. (2001). Efficacy and safety of recombinant human activated protein C for severe sepsis. N. Engl. J. Med..

[26] Abraham E., Laterre P.F., Garg R., Levy H., Talwar D., Trzaskoma B.L. (2005). Drotrecogin alfa (activated) for adults with severe sepsis and a low risk of death. N. Engl. J. Med..

[27] Bernard G.R., Margolis B.D., Shanies H.M., Ely E.W., Wheeler A.P., Levy H. (2004). Extended evaluation of recombinant human activated protein C United States Trial (ENHANCE US): A single-arm, phase 3B, multicenter study of drotrecogin alfa (activated) in severe sepsis. Chest.

[28] Abraham E., Reinhart K., Opal S., Demeyer I., Doig C., Rodriguez A.L. (2003). Efficacy and safety of tifacogin (recombinant tissue factor pathway inhibitor) in severe sepsis: a randomized controlled trial. JAMA.

[29] Jaimes F., De la Rosa G., Arango C., Fortich F., Morales C., Aguirre D. (2006). A randomized clinical trial of unfractioned heparin for treatment of sepsis (the HETRASE study): Design and rationale [NCT00100308]. Trials.

[30] Jaimes F., De La Rosa G., Morales C., Fortich F., Arango C., Aguirre D. (2009). Unfractioned heparin for treatment of sepsis: A randomized clinical trial (The HETRASE Study). Crit. Care Med..

[31] Yamaguchi T., Kitajima Y., Miyauchi Y., Izawa K., Tanaka M., Hirata M. (2018). Assessment of bleeding in patients with disseminated intravascular coagulation after receiving surgery and recombinant human soluble thrombomodulin: A cohort study using a database. PLoS ONE.

[32] Singer M., Deutschman C.S., Seymour C.W., Shankar-Hari M., Annane D., Bauer M. (2016). The Third International Consensus Definitions for Sepsis and Septic Shock (Sepsis-3). JAMA.

[33] Gando S., Levi M., Toh C.H. (2016). Disseminated intravascular coagulation. Nat. Rev. Dis. Primers.

[34] Hato T., Maier B., Syed F., Myslinski J., Zollman A., Plotkin Z. (2019). Bacterial sepsis triggers an antiviral response that causes translation shutdown. J. Clin. Invest..

[35] Cohen J. (1992). A Power Primer. Psychol Bull..

[36] Napolitano L.M. (2018). Sepsis 2018: Definitions and Guideline Changes. Surg Infect..

