# Supplementary material for: Human Soluble Recombinant Thrombomodulin, ART-123, Resolved Early Phase Coagulopathies, but Did Not Significantly Alter the 28 Day Outcome in the Treatment of DIC Associated with Infectious Systemic Inflammatory Response Syndromes
Source: J Clin Med. 2019 Sep 27;8(10):1553. doi: 10.3390/jcm8101553 (PMC6832475; doi:10.3390/jcm8101553)
Supplement: Supplementary file 1 [file jcm-08-01553-s001.pdf]

Supplementary Information for

Human Soluble Recombinant Thrombomodulin, ART-123, Resolved Early Phase Coagulopathies, but Did Not Significantly Alter the 28 Day Outcome in the Treatment of DIC Associated with Infectious Systemic Inflammatory Response Syndromes

Shusuke Mori *, Tomohiko Ai, Toshiki Sera, Kanae Ochiai and Yasuhiro Otomo

Supplementary Table 1

Table S1. International Society on Thrombosis and Hemostasis (ISTH) score.

	**Point**
Platelet count (/μL)	
>100,000	0
50,000–100,000	1
<50,000	2
Fibrin-related marker	
No change	0
Moderate rise	2
Strong rise	3
Prothrombin time prolongation (s)	
≤3	0
>3 but <6	1
≥6	2
Fibrinogen (g/L)	
>1	0
<1	1
DIC is not overt	0–4
DIC is probable	5–8
